# Composite quality measures of abdominal surgery at a population level: systematic review

**DOI:** 10.1093/bjsopen/zrad082

**Published:** 2023-11-01

**Authors:** Joel Rajesh, Jan Sorensen, Deborah A McNamara

**Affiliations:** Healthcare Outcomes Research Centre (HORC), Royal College of Surgeons in Ireland (RCSI), Dublin, Ireland; Healthcare Outcomes Research Centre (HORC), Royal College of Surgeons in Ireland (RCSI), Dublin, Ireland; National Clinical Programme in Surgery (NCPS), Royal College of Surgeons in Ireland (RCSI), Dublin, Ireland; Department of Colorectal Surgery, Beaumont Hospital, Dublin, Ireland

## Abstract

**Background:**

Measurement of surgical quality at a population level is challenging. Composite quality measures derived from administrative and clinical information systems could support system-wide surgical quality improvement by providing a simple metric that can be evaluated over time. The aim of this systematic review was to identify published studies of composite measures used to assess the overall quality of abdominal surgical services at a hospital or population level.

**Methods:**

A search was conducted in PubMed and MEDLINE for references describing measurement instruments evaluating the overall quality of abdominal surgery. Instruments combining multiple process and quality indicators into a single composite quality score were included. The identified instruments were described in terms of transparency, justification, handling of missing data, case-mix adjustment, scale branding and choice of weight and uncertainty to assess their relative strengths and weaknesses (PROSPERO registration: CRD42022345074).

**Results:**

Of 5234 manuscripts screened, 13 were included. Ten unique composite quality measures were identified, mostly developed within the past decade. Outcome measures such as mortality rate (40 per cent), length of stay (40 per cent), complication rate (60 per cent) and morbidity rate (70 per cent) were consistently included. A major challenge for all instruments is the reliance of valid administrative data and the challenges of assigning appropriate weights to the underlying instrument components. A conceptual framework for composite measures of surgical quality was developed.

**Conclusion:**

None of the composite quality measures identified demonstrated marked superiority over others. The degree to which administrative and clinical data influences each composite measure differs in important ways. There is a need for further testing and development of these measures.

## Teaser

We conceptualize composite quality measures as a sliding scale where the optimum balance between clinical and administrative inputs varies according to the purpose of assessment. Better validation of composite quality measures requiring less clinical input could provide a cost-effective tool that can act as a signal for population-level changes in surgical quality.

## Introduction

Equitable access to high-quality surgery should be a population health priority, but its provision is impacted by trade-offs and policy choices that sometimes have unanticipated consequences. The impact of population-level policy decisions, like the centralization of surgical cancer services^1^, on the quality of surgical services generally is rarely evaluated. The ‘whole of population’ context is a distinct perspective that often differs from the point of view of individual surgeons and departments.

The lack of consensus about how surgical quality is defined at a population level has important implications in the implementation and evaluation of healthcare policy. While this subject is of less relevance to individual practicing surgeons and departments, the absence of consensus means it is difficult to identify the positive and negative impacts on surgery as a whole, when changes in national budgetary resource allocation or configuration of services are implemented. Existing literature focuses largely on procedure-specific classification of adverse outcomes^2^ as well as on benchmarking^3^ for the purposes of quality assurance and commissioning. The distinction between data for quality assurance and quality improvement is well described^4,5^, but validated and objective measures to support hospital or population-level quality improvement in surgery remain sparse. National improvement programmes in surgery are often confined to specialties^5^ (for example Getting It Right First Time (GIRFT)^6^) or conditions such as cancer^1^, are typically resource intensive to implement (for example the National Emergency Laparotomy Audit)^6–9^, prioritize structure and process measures^10^, and are at risk of being confounded by random-cause variation when volumes are low^11^. Comparisons between hospitals are challenging especially when there are variations in case-mix and volume^11^, but referral patterns and case-mix tend to be stable over time^12^. While the measurement of surgical quality in healthcare systems remains difficult, population-level measures to evaluate the impacts of policy changes on the overall quality of surgical care would enhance the ability of surgical leaders to advocate for surgical care.

From a technical perspective, identification of a simple, validated and reliable measure of surgical quality based on administrative data offers several putative advantages. It is reproducible, reduces surveillance bias^13,14^ and is less vulnerable to changes in coding practice as administrative data is routinely collected independently of individual surgeons or departments^15^. Additionally, development of low-cost composite quality measures is important if publicly funded healthcare systems are to reduce the administrative burden of data collection in surgery^16^ and increase the value of surgical care to meet the increasing needs of the population. Despite clear limitations when composite measures are used to compare hospitals treating different populations and case-mix^11^, monitoring of a validated composite quality measure over time may be a useful component of a population-level quality assessment system for surgery, if it can act as a signal to identify impacts of population-level changes or variation that may require deeper evaluation.

The aim of this research was to systematically review published studies of composite quality measures that may be used to assess the overall quality of abdominal surgery at a population level. The primary outcome of interest was to identify composite quality measures that may be suitable for use with data on emergency abdominal surgery from existing population-level administrative systems.

## Methods

### Search strategy

This systematic review was registered with the International Prospective Register of Systematic Reviews PROSPERO (CRD42022345074) on 9 July 2022. The review was conducted according to the PRISMA guidelines^17^. The search strategy aimed to identify published studies developing or using measurement instruments based on administrative and clinical data systems to assess the overall quality of abdominal surgery. Measurement instruments for patient-reported outcomes were excluded. These instruments are frequently composite scores combining several quality indicators into a single score. An initial limited scoping search using MEDLINE, Embase and Scopus was undertaken to identify articles on the topic. There was no year restriction. The final search strategy, including all identified keywords and index terms from the initial search, was adapted for the final search in PubMed and MEDLINE, which were chosen over the other data sources as they provided more relevant additions during the initial scoping search. The focus of the systematic review was measurement instruments (composite quality measures) that evaluated the overall quality of abdominal surgery based on data from population-level administrative and clinical data systems. The goal was to critically assess how the measurement tools captured various aspects of surgical quality. Titles and abstracts were screened using Rayyan AI™. We excluded any study of non-abdominal or non-gastrointestinal surgery^18^. At each stage, any conflicts or uncertainties were resolved by discussion with all authors. Full details of the search strategy are listed in *Appendix S1*.

### Study review

The full text of each manuscript meeting the inclusion criteria was downloaded for further scrutiny by all authors. The data extracted included publication data (authors, publication year, country or region of study, study design, sample size and setting), population data (for example patient characteristics, surgical procedures, age range) and specific details about the composite quality measure used or developed (name of the measure, its purpose, the number and type of variables included, range of score, perspective, scoring methods and weighting, length of follow-up, calibration, application, validation methods). This data was imputed into summary tables. Included studies were assessed using the items proposed by Barclay *et al*^11^. Study quality was assessed using the Critical Appraisal Skills Programme (CASP) appraisal checklist. Reasons for the exclusion of full-text manuscripts that did not meet the inclusion criteria (*Appendix S2*) were recorded.

### Interpretation

Similarities and differences between the included composite measures, especially the spectrum of administrative and clinical data points used by each composite measure, were compared and synthesized. Three categories, ranging from ‘minimal’ to ‘moderate’ to ‘significant’ clinical input were defined and used to evaluate the relative strengths and weaknesses of each composite measure. A detailed analysis of the perspective from which data was collected in the construction of each composite measure, as well as associated temporal and cost factors, was undertaken and summarized. Following analysis of the literature, a conceptual framework for composite measures of surgical quality at a population level was defined.

## Results

**Fig. 1:**
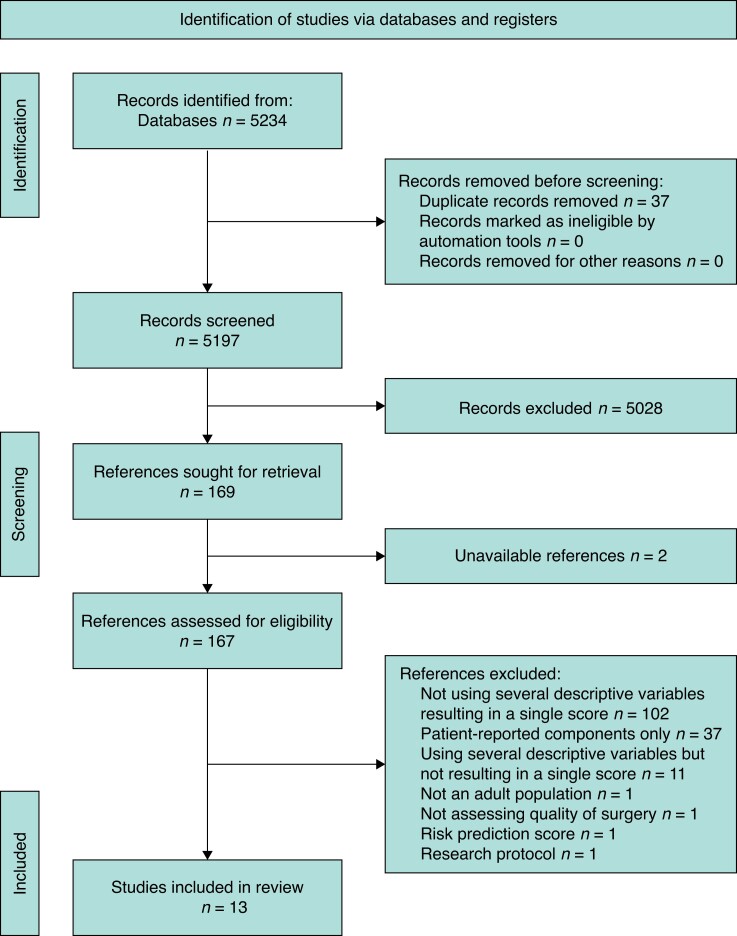
PRISMA diagram with systematic review of composite quality measures for surgical outcomes

In the initial search, 5197 articles were identified after the exclusion of duplicates. A PRISMA flow diagram is shown in *Fig. 1*. One hundred and sixty-nine articles underwent full-text review. The full-text review identified 13 studies meeting all full inclusion and exclusion criteria.

### Study quality

From 13 included studies, 10 unique composite quality measures were identified. The studies were contemporary with six studies published in the last 5 years and 11 within the last decade. All studies used retrospective data. Some studies used data from clinical trials, physician-reported or health-professional reported outcomes, and patient-reported outcomes (PROMs^19,20^) but those exclusively reporting PROMs were excluded from this review. All included studies were based on data for more than 100 patients. *Table 1* provides a detailed overview of each of the composite quality measures identified. *Table 2* provides an analysis of the methodology of the development of each composite measure. The authors’ interpretation of factors influencing the utility of each composite measure for the intended purpose of population-level assessment of surgical quality in terms of necessary clinical and administrative inputs is summarized in *Tables 3* and *4*.

**Table 1:** Detailed information on each outcome measure

Score name	Objective of instrument	Included variables	Range of score	Weight of variables	Length of follow-up	Data used to calibrate	Relevance of variables	Validation	Examples of application
Days Alive and Out of Hospital^21^ (Canada)	Easily calculated quality measure of patient outcomes after surgery. Associated with patient characteristics, surgical complexity, in-hospital complications and longer-term outcomes.	Mortality Duration of hospital stay Readmission within 30 days.	0–30 Higher score indicates worse outcome as reflects longer duration of hospital stay. Patient death automatically scores as 0 Every point on scale considered important.	All variables combined with equal weighting.	Looks at listed variables between date of the index surgery and 30th postoperative day.	Association with characteristics: Patient (age, sex, co-morbidities). Hospital (academic status, total bed number, surgical volume at each institution). Surgical (procedure types, procedure duration).	Duration of hospital stay is a surrogate for quality and speed of recovery after surgery. Readmission is a surrogate for postoperative complications.	Validated in sample of 540 072 patients. Construct validity assessed. Hierarchical multivariable quantile regression model used to assess association of patient, surgical and hospital characteristics with score.	Perioperative clinical trials. Major elective non-cardiac surgical procedures.
I-FEED^22^ (Canada)	An outcome measure for postoperative ileus (prolonged absence of bowel function after surgery).	Severity of limitation of oral intake, vomiting, physical examination, symptom duration. 0–2 considered normal 3–5 considered intolerance 6+ considered dysfunction	0–6+ Higher score indicates worse GI dysfunction.	Intake: 3 points Nausea: 3 points Emesis: 3 points Exam: 3 points Duration of symptoms: 2 points.	Daily scores generated up to hospital discharge or day 7.	Confirmed four main hypotheses, association of score with: Longer time to GI motility Longer length of hospitalization More complication patient-reported recovery.	Classification developed by expert consensus to account for clinically relevant aspects of GI recovery, factors that influence management and levels of dysfunction associated with increased complications and cost.	Validated in sample of 128 patients. Construct validity for score to measure the construct of postoperative GI recovery was tested according to the four main hypotheses (GI motility, length of stay, complications, patient-reported quality of recovery).	Laparascopic colorectal surgery
Hospital stay, readmission and mortality rate^3,23–25^ (United States)	Easily calculated quality measure of patient outcomes after surgery.	Mortality Duration of hospital stay Readmission within 30 days Elective or emergent status	0–10 Higher score indicates worse outcome. Every point on scale considered important.	LOS (6 categories): 5 points Mortality rate: 5 points Readmission: 1 point	30 days	Correlation between the hospital-level complication rate and HARM scores used for internal validation.	Previous studies have shown the value of each individual HARM component as a measure of quality.	Validated in a sample of 81 622 colectomy discharges; of which 44% were emergent. Logistic regression showed that the complication rate was significantly associated with each HARM component. Reliability and validity assessed through bootstrapping correlation coefficients.	Colorectal surgery
Surgical complication OUTcome^26^ (United States)	Easily calculated quality measure of patient outcomes after surgery with focus on the severity of postoperative complications.	Complication severity Mortality 0 indicates no complications 100 indicates death	Minimum score 0, no upper limit Higher scores indicate worse outcome.	Points based on severity (‘grade’) of complication. No pre-set guidelines for each grade, that is points assigned to a particular grade vary by type of complication.	Score generated during each day of hospitalization if a complication meeting criteria arose.	Not described	Classification developed by expert consensus. A panel of surgeons assigned a SCOUT severity score for each grade of complication.	Trialled in a sample of 9000 general and vascular surgical cases. Statistical validation methods unclear.	General and vascular surgery
Mortality, transfer, length of stay^27^ (German)	Easily calculated quality measure of patient outcomes after surgery.	Postoperative mortality rate. Postoperative transfer to another hospital. Duration of hospital stay above predefined duration.	Positive (MTL+) or negative (MTL−). Positive MTL indicates worse outcomes.	Any one of the variables occurring results in an overall positive score.	Length of follow-up varies, but 30 days or 22 days suggested.	Association with characteristics: Patient (such as ASA, complications, age, tumour-dependent factors).	MTL+ has a high correlation with existing patient risk factors and strongly correlated with occurrence of postoperative complications.	Trialled in sample of 14,978 patients undergoing colorectal resection. MTL rates calculated and compared to well established single outcome measures using multivariate regression analysis. For each outcome measure, postoperative complications were tested regarding predictability	Colorectal cancer surgery
Textbook Outcome^28^ (Netherlands)	Composite quality measure of clinical process indicators that measures if a series of predefined desirable short-term health outcome indicators are met	Different positive short-term health outcomes based on procedure.	Positive or negative. Positive TO indicates better outcomes.	All variables must occur to result in an overall positive score.	Not applicable. Review of existing database.	Indicators of good clinical outcome selected after literature review. Justification for each selection provided in *Appendix S1.*	Unsure about extent that medical complexity and co-morbidity rate variation influence TO scores.	Trialled in sample of 45,848 patients undergoing range of surgical procedures. To assess impact of clinical indicators where the total TO was not met (TO = 0), the specificity of each indicator was determined. A pairwise comparison between TO score on hospital level and score per indicator was performed per treatment.	Gastrointestinal diagnoses requiring endoscopic intervention. Can be adapted for any procedure.
Postoperative Morbidity Index^29^ (United States)	A measure used to estimate both the overall frequency and severity of complications in a postoperative population.	Complication severity and number of patients. Score is sum of complication severity weights divided by total number of patients.	0–1.00 Higher score indicates worse outcome. Score of zero indicates that no patient had a postoperative complication. Score of 1.00 indicates that every procedure in the series resulted in a postoperative death. Every point on scale considered important	Each complication individually weighted based on severity. Severity calculated by expert consensus.	Not applicable. Review of existing database	Relevant complications selected through the validated and commonly used ACS-NSQIP system.	Severity of complications assigned using the validated Accordion Severity Grading System.	Trialled in sample of 655 patients undergoing distal pancreatectomy. Standard deviation of score was calculated as the weighted mean of standard deviations of institutional score values. Patient severity weight (0–1.00) was used as the dependent variable in regressions seeking correlates to score. Categorical variables were tested using two-sided independent sample *t* tests and ANOVA. For continuous variables, univariate linear regression was used.	Distal pancreatectomy
Therapeutic Intervention Scoring System^30^ (Germany)	A comprehensive outcome measure for postoperative patients in surgical ICU. Intermediate TISS score available for postoperative patients not in ICU.	Comprehensive list of variables including basic activities of care, ventilatory and renal support, cardiovascular support, and neurologic, interventions and metabolic support.	Varies	Each component in the score given equal weighting.	Scores calculated during hospital stay until discharge.	Initial simplified TISS28 developed based on analysis of 10 079 ICU records	An increased level of therapeutic activities at the end of ICU stay is associated with worse hospital outcome; 21.4% of patients with TISS of 20 or greater on discharge died subsequently during hospital stay.	Trialled in sample of 1808 patients in a surgical ICU. Some statistical tests not applied to avoid arbitrary significant results based on the large number of cases rather than on clinically relevant differences. Stepwise logistic regression analysis applied to evaluate score on the day of admission for prediction of hospital mortality rate.	Primarily used in an ICU setting where connected monitors can collect data.
Patient Quality Score^31^ (United States)	A comprehensive outcome measure assessing adherence to a comprehensive set of perioperative process-based Quality Indicators (QIs).	Comprehensive outcome list examining process-based QIs and complications: Prophylactic antibiotics, postoperative euglycaemia, prophylactic venous thromboembolism therapy, central venous line, urinary catheter, postoperative ambulation, medication list, pressure ulcer risk assessment, oral intake documentation, surgical safety checklist.	1–100% The patient quality score was calculated for each patient as the number of QIs passed divided by the number of QIs for which each patient was eligible.	Unclear	Not applicable. Review of existing database.	A Delphi consensus survey was used to determine QIs most relevant to the patient population. Inter-rater agreement was assessed for each QI using per cent agreement and the AC1 statistic.	Not described	Trialled in sample of 273 patients undergoing abdominal surgery. A Poisson regression used to test for association between patient quality score and occurrence of complications, which was adjusted for other patient characteristics. Poisson regression revealed that as quality score increased, incidence of postoperative complications decreased. Sensitivity analysis revealed that association was likely driven by postoperative ambulation QI.	Elective major abdominal operations.
DIMICK *et al*. score^32^ (United States)	A composite quality measure that incorporates information from multiple quality indicators to optimally predict ‘true’ risk-adjusted morbidity rate for each operation.	Morbidity rate, including morbidity rate with other related procedures. Duration of hospital stay. Re-operation rate	Hospitals ranked based on composite quality measures into 1-star, 2-star or 3-star rating.	The weight on each quality indicator is determined for each hospital to minimize the expected mean squared prediction error, using an empirical Bayes methodology. Weight based on the hospital-level correlation of each quality indicator with the mortality rate, and the reliability with which each indicator is measured.	Not applicable Review of existing database.	Calculated the correlation of each individual quality indicator with the mortality rate and calculated the average reliability of the standardized mortality rate and complication ratios for each procedure.	Adding risk-adjusted morbidity rates with ‘other’ procedures enhanced the reliability of hospital performance assessment. The ability to ‘borrow’ signals from these other operations reflects the presence of shared structure and process that lead to better outcomes.	Validated in a sample of patients undergoing aortic valve replacement. Estimated random-effect logistic models of mortality rate at the patient level, controlling for the same patient covariates. The random-effect logistic model used. Constructed an R-squared statistic for the 2002 to 2003 forecast equal to the amount of variation being predicted by the composite quality measure as percentage of all hospital-level variation.	Ventral hernia repair. Colon resection. Lower extremity bypass surgery. Abdominal aortic aneurysm repair. Aortic valve replacement.

GI, gastrointestinal; LOS, length of (hospital) stay; HARM, Hospital stay, Readmission, and Mortality; SCOUT, Surgical Complication OUTcome; MTL, Mortality, Transfer, Length-of-stay; TO, Textbook Outcome; NSQIP, National Surgical Quality Improvement Program; TISS, Therapeutic Intervention Scoring System; I-FEED, Intake, response to nausea treatment, Emesis, Exam, and Duration; ACI, first-order agreement coefficient; DIMICK, Dimick et al. 2013.

**Table 2:** Analysis of the methodology of development of each composite quality measure

Instrument	Transparency in calculation^11^	Justified selection of individual measures^11^	Handling missing measure information^11^	Handling missing measure information^11^	Banding onto scales^11^	Justification for weights^11^	Justification for weights^11^	Uncertainty^11^
	Are all important methodological details easily accessible in a public document?	Are the measures used equally applicable across all rated hospitals?	Is missing measure information handled in a way that can introduce bias?	Are component measures adequately adjusted for case-mix?	Are measures standardized using banding?	Is there an apparent justification for the weights used?	Is any sensitivity analysis of the choice of weights reported?	Is the uncertainty in the final composite rating presented?
DAOH^21^	Yes	Yes. Insufficient clarity on process by which decisions made to choose measures. Length of stay dominates	Patients with missing data not included	Not discussed	Yes, mortality rate and length of stay banded onto a scale	Insufficient justification for calculations used	Yes	No
I-FEED^22^	Yes, however measures have a subjective element	Yes. Measures are related to procedure but insufficient justification for selection	No information	Not discussed	Yes, measures banded onto an arbitrary scale	Yes, developed by expert consensus	Yes	No
HARM^3,24–25^	Yes	Yes Insufficient clarity on process by which decisions made to choose measures	Patients with missing data not included	Not discussed	Yes, measures banded onto an arbitrary scale	LOS scaled based on normal distribution curve. No justification for other weights	No	No
SCOUT^26^	Yes, however measures have a subjective element	Yes. Measures chosen from existing list of complication types	Information collected for score	Not discussed	Yes, complications banded into four grades to get measure onto a consistent scale	Yes, developed by expert clinical opinion	No	Partially
MTL^27^	Yes	Yes. Insufficient clarity on process by which decisions made to choose measures	Patients with missing data not included	Authors state that analysis is not adjusted for case-mix	Yes, length of stay banded onto a scale	No	No	No
TO^28^	No, although measures included in this study are clear—there is no clear consensus of what measures should be included in future studies	No, measures may not be equally applicable to some hospitals	Hospitals without relevant data not included	Yes	Yes, measures banded into positive or negative result	Not applicable	No	No
TISS^30^	Yes	No, measures may not be equally applicable to some hospitals	Information collected for score	Not discussed	Yes, measures banded onto an arbitrary scale.	Yes, intensity of involvement	No	No
PQS^31^	Yes	Yes	Hospitals without relevant data not included	Not discussed	Yes, each measure banded as one ‘QI’	Yes, clinical opinion	Yes	Partially
DIMICK^32^	Yes, but in another paper. Insufficient clarity on how data from ‘other’ procedures incorporated	Yes	Hospitals without relevant data not included	Yes	No	Yes, well justified	Yes	Partially
PMI^29^	Yes	Yes	Hospitals without relevant data not included	Not discussed	Yes, severity of complications banded using Accordion Severity Grade	Yes, partially uses previously validated grading system	No	No

LOS, length of (hospital) stay; HARM, Hospital stay, Readmission, and Mortality; DAOH, Days Alive and Out of Hospital; SCOUT, Surgical Complication OUTcome; MTL, Mortality, Transfer, Length-of-stay; TO, Textbook Outcome; NSQIP, National Surgical Quality Improvement Program; TISS, Therapeutic Intervention Scoring System; I-FEED, Intake, response to nausea treatment, Emesis, Exam, and Duration; PQS, Patient Quality Score; DIMICK, Dimick et al. 2013; PMI, Post-operative Morbidity Index; QI, quality indicator.

**Table 3:** Strengths and weaknesses assessment of each composite quality measure

	Instrument	Strengths	Weaknesses
Minimal clinical input	DAOH^21^	Simple to calculate. Uses data points that are routinely collected and available. Similar approach to HARM and MTL	Simplistic approach. Includes variables on mortality rate and length of stay but ignores other quality indicators
	HARM^3,23–25^	Simple to calculate. Uses data points that are routinely collected and available. Uses more data points than DAOH. Similar approach to MTL and DAOH	Simplistic approach. Includes variables on length of stay, readmission and mortality rate but ignores other quality indicators
	MTL^27^	Simple to calculate and uses data points that are routinely collected and available. Uses more data points than DAOH. Similar approach to HARM and DAOH	Simplistic approach. Includes variables on mortality rate, transfer to another hospital and length of stay but ignores other quality indicators
Moderate clinical input	TO^28^	Can be tailored to suit any procedure. Quality indicators chosen by expert opinion	Simplistic approach which assumes all selected short-term outcomes have equal importance. Subjective. May require more data than routinely collected and available
	PQS^31^	Simple to calculate. Data points are routinely collected and available. Assesses more quality indicators (10) than most other scores. Quality indicators chosen by Delphi consensus survey	May require more data than routinely collected and available even though it is designed to be used with existing records
	DIMICK^32^	Uses data points that are routinely collected and available. Utilizes quality information from other related procedures to improve precision of quality measurement for each operation. Weights are calculated for each quality indicator to improve precision	Dependence on a database collected by others. Less simple to calculate, requires statistical support
	PMI^29^	Data points are routinely collected and available. Incorporates already validated grading systems	Dependence on a database collected by others. Less simple to calculate, may require statistical support.
Significant clinical input	SCOUT^26^	Detailed analysis using many different data points. Quality indicators chosen by expert opinion	Requires manual collection of the outcome metrics used
	I-FEED^22^	Detailed analysis using many different data points	May be expensive and time-consuming to run. Ileus only one relevant outcome. Requires expertise
	TISS^30^	Detailed analysis using many different data points. Has been widely used	Requires ICU-level equipment which can automatically collect vast amounts of data. Requires expertise

LOS, length of (hospital) stay; HARM, Hospital stay, Readmission, and Mortality; DAOH, Days Alive and Out of Hospital; SCOUT, Surgical Complication OUTcome; MTL, Mortality, Transfer, Length-of-stay; TO, Textbook Outcome; NSQIP, National Surgical Quality Improvement Program; TISS, Therapeutic Intervention Scoring System; I-FEED, Intake, response to nausea treatment, Emesis, Exam, and Duration; PQS, Patient Quality Score; PMI, Post-operative Morbidity Index; DIMICK, Dimick et al. 2013.

**Table 4:** Clinical *versus* administrative comparison of each composite quality measure

Instrument	Procedure/specialty dependent *versus* system relevance	Time perspective	Routine/research based	Cost of obtaining data	Degree of clinical expertise required
DAOH^21^	System	Up to 30 days after index surgery	Routine	Low	None
I-FEED^22^	Procedure/specialty	Up to discharge or POD 7	Research	High	High
HARM^3,23–25^	System	Duration of hospital stay and 30-day readmission and mortality rate	Routine	Low (High if relying on ACS-NSQIP)	None
SCOUT^26^	Procedure/specialty	Length of postoperative stay and 30-day mortality rate	Research	High	High
MTL^27^	System	Duration of hospital stay and 22- or 30-day mortality rate	Research	Low (High if relying on ACS-NSQIP)	None
TO^28^	Procedure/specialty	Length of postoperative stay	Research	Variable	Variable
TISS^30^	Both	Length of postoperative stay	Routine ICU	High	Variable
PQS^31^	Procedure	Length of postoperative stay	Routine	Variable (High if relying on ACS-NSQIP)	Variable
DIMICK^32^	System	Duration of hospital stay and 30-day morbidity rate and mortality rate	Routine	Low (High if relying on ACS-NSQIP)	None
PMI^29^	Procedure/specialty	Up to 30 days after index surgery	Routine	Low (High if relying on ACS-NSQIP)	Variable

LOS, length of (hospital) stay; HARM, Hospital stay, Readmission, and Mortality; DAOH, Days Alive and Out of Hospital; SCOUT, Surgical Complication OUTcome; MTL, Mortality, Transfer, Length-of-stay; TO, Textbook Outcome; NSQIP, National Surgical Quality Improvement Program; TISS, Therapeutic Intervention Scoring System; I-FEED, Intake, response to nausea treatment, Emesis, Exam, and Duration; PQS, Patient Quality Score; PMI, Post-operative Morbidity Index; DIMICK, Dimick et al. 2013; POD, post-operative day; NSQIP, National Surgical Quality Improvement Program

### Measures selected

Among the identified composite quality measures, seven (70 per cent) included some measure of morbidity rate, four (40 per cent) included a measure of postoperative mortality rate, while four (40 per cent) included duration of hospital stay and four (40 per cent) included variables specific to the procedure being investigated.

The ‘postoperative Mortality rate, postoperative Transfer to other hospital, postoperative Length of stay’ (MTL) is a composite quality measure that can be derived from routine administrative data. It includes variables on mortality rate, transfer to another hospital and length of stay. This study shows that the MTL measure has a better ability to discriminate between hospital surgical quality compared with a single quality indicator, even with low hospital caseloads or low ‘event’ occurrence rates of each outcome measure^27^.

The ‘Hospital stay, Readmission, and Mortality’ (HARM) measure is a composite quality measure using data on mortality rate, readmission and total length of stay to compare different surgeons and hospitals. HARM scores are calculated for each discharge with the formula; HARM = Length of stay (LOS) category (0–5) + discharge status (0/1) × 5 + readmission (0/1). Pearson correlation coefficients between the hospital-level complication rate (including postoperative infection, haemorrhage, wound dehiscence, peritonitis/anastomotic leak and other gastrointestinal complications) and HARM scores were used for internal validation, showing that the HARM score was correlated with clinical outcomes. However, the correlation was more apparent in the patients undergoing elective surgery than emergency surgery^3,23–25^.

The ‘Textbook Outcome’ method of composite quality measurement is different from the other scores as it focuses on assessing whether all predefined positive short-term outcomes have been met, rather than assessing the rate of negative events. This list of ideal positive short-term outcomes varies between procedures and between studies^28^.

The ‘Days Alive and Out of Hospital’ (DAOH) is a composite measure that incorporates the duration of hospital stay, additional stays resulting from readmissions and mortality rate. This measure has been validated in a cohort of emergency laparotomy patients. DAOH is calculated by identifying the number of days spent in hospital, including initial and any subsequent hospital stays, and subtracting this sum from the total interval length, using defined intervals of 30, 90, 180 or 365 postoperative days. If patients die within the defined period, they receive a DAOH score of 0. As a result, 0 is the worst possible outcome with increasing numbers indicating the more desirable outcomes of the greatest possible number of days alive and out of hospital^21,33,34^.

The ‘Intake, Feeling nauseated, Emesis, Exam, Duration of symptoms' (I-FEED) measure is a composite quality measure specifically designed to measure recovery after gastrointestinal surgery. It uses five elements based on detailed clinical inputs (oral intake, response to nausea treatment, emesis, exam and duration) scoring each either 0, 1 or 3 points. The cumulative score classifies return of postoperative function into three categories: normal, postoperative gastrointestinal intolerance and postoperative gastrointestinal dysfunction^22^.

The ‘Surgical Complication Outcome’ (SCOUT) measure is a composite measure using predefined lists of clinically significant postoperative complications. Examples of complications relevant to gastrointestinal surgery include perforation, gastrointestinal bleeding, ileus and anastomotic leak whereas those specified for general surgery include drug reactions, injury to adjacent structures and intra-abdominal abscess. Complications are scored with a ‘grade’ of severity based on their consequences. For example, colon ischaemia requiring clinical observation alone receives a score of 31, sepsis or ICU admission are assigned a score of 50 and death is assigned a score of 100. A high degree of clinical input to this measure is therefore required^26^.

The ‘Postoperative Morbidity Index’ (PMI) is a composite measure which incorporates complication severity and the total number of patients affected. Complication severity is assigned using the Accordion Severity Weighting System. While PMI was designed as a measure of morbidity rate more than a composite quality measure, it may still have value as a measure for the quality of surgical care^29^.

The ‘Patient Quality Score’ (PQS) is a composite quality measure which measures the quality of surgical care by calculating adherence to 10 process-based quality indicators (PQIs) for each patient. This is calculated as the proportion of number of PQIs passed in relation to the number of PQIs eligible. These PQIs are more specific than the more general metrics used by other composite measures, with examples including whether a patient received prophylactic antibiotics and whether a pressure ulcer risk assessment was performed^31^.

The ‘Therapeutic Intervention Scoring System’ (TISS) incorporates therapeutic, diagnostic and nursing activities to assess the quality of care received. TISS-28 incorporates a list of 28 variables into the measure, including basic activities of care, ventilatory support, cardiovascular support, renal support, neurologic support, metabolic support and specific interventions^30^.

The DIMICK measure is a composite quality measure that incorporates several quality indicators (morbidity rates, reoperation, length of stay) along with morbidity rate for other related procedures performed. This composite measure differs from others in that it ‘borrows’ quality metrics from related procedures based on the assumption that better outcomes for related procedures reflect the presence of shared structures and processes that predict better outcomes for all surgical procedures^32^.

### Measures validation and assessment

The analysis of included composite quality measures is summarized in *Table 2*. Despite generally good descriptions of how each composite quality measure was validated, many studies provided only a brief, and sometimes superficial, overview of the process. The DAOH manuscript reports analysis to determine the association of the measure with patient, surgery and hospital-level characteristics^21,33^. Both I-FEED and SCOUT were developed based on expert consensus but only I-FEED reports evidence of construct validity^22,26^. The authors of HARM and MTL developed their measures through trials using existing inpatient databases. Each measure was correlated with complication rates and other outcome measures to test validity^23–25,27^. The authors of Textbook Outcome (in the gastrointestinal context) selected their ideal outcomes through literature search and tested the instrument with data from a benchmark database^28^. The authors of PMI combined two existing validated systems to develop their score^29^. The TISS system, first described in 1974, was subsequently simplified based on detailed analysis of a research database^30^. The PQS utilized a Delphi consensus survey to select their included quality indicators for their score and tested the association between the score and the occurrence of complications^31^. The authors of the DIMICK measure developed it by combining quality indicators found in the American College of Surgeons National Surgical Quality Improvement Program (ACS-NSQIP). This measure was validated by assessing how the measure for 1 year predicated morbidity rate for the next year^32^.

*Table 3* provides a general overview of strengths and weaknesses attributed to each composite quality measure. The authors of DAOH acknowledged greater sensitivity to patient and surgery-level characteristics than to variation in hospital characteristics^21,33^. I-FEED is self-recognized to be more useful as a research tool than in clinical practice^22^. The authors of HARM and MTL identified each may lack potentially important variables^23–25,27^. SCOUT is identified as a tool that can complement other existing measures of mortality rate and morbidity rate but may have less value by itself^26^. The authors of Textbook Outcome acknowledge the underlying weakness that hospitals being compared should have comparable medical complexity and case-mix^28^. PMI has a key weakness arising from its reliance on accurate complication reporting^29^. The TISS system, while widely used, has been criticized for being time-consuming, poorly defined, inconsistently modified, incomplete and outdated^30^. The authors of PQS acknowledge only the face validity of the quality indicators was established^31^. The authors of the DIMICK measure acknowledge a lack of inputs based on hospital characteristics^32^.

### Interpretation and perspective

**Fig. 2:**
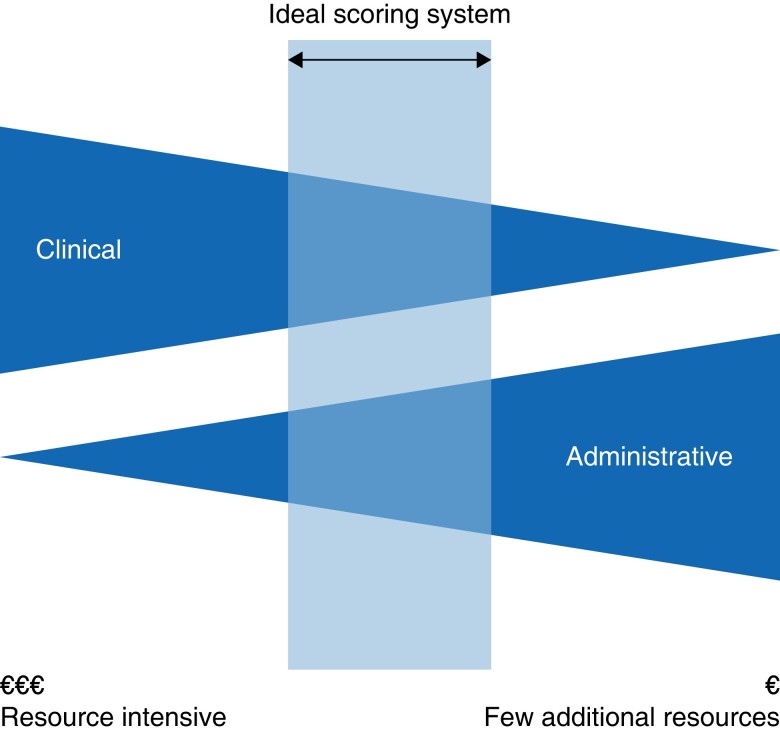
A conceptual framework for composite outcome measures of surgical quality. The ‘ideal’ scoring system will vary depending on the underlying administrative data, clinical expertise and available resources

The amount of clinical input required to calculate a composite measure was identified as a key constraint in the utility of the measure for evaluation of surgical quality at a population level, *Table 1*. The strengths and weaknesses of each composite measure accordingly are shown in *Table 3*. A detailed analysis of the perspective from which data was collected in the construction of each composite measure, as well as associated temporal and cost factors, is summarized in *Table 4*. Following analysis of the literature, a conceptual framework for composite measures of surgical quality at a population level was defined (*Fig. 2*).

Most composite quality measures used data derived from routinely collected healthcare databases, with only one measure (TISS) using physiologic data acquired by medical equipment or sensors. The I-FEED measure combined patient-reported and staff-reported data into a composite measure, whereas others used only staff-reported data. Even for measures that were found to be valid and reliable, their dependence on databases routinely or additionally collected by healthcare staff has implications for the reliability and reproducibility of results. *Table 4* provides a comparison of the clinical *versus* administrative characteristics of each composite quality measure.

Although this review is limited to composite quality measures used in abdominal surgery, most measures identified either have the potential to be adapted for use or have already been used in cohorts with other patients. The DAOH, HARM, MTL and DIMICK measures could feasibly be used for different types of surgery without modification. The SCOUT and PMI measures could be used for other types of surgery if new complication severity grades were specified for each procedure. The Textbook Outcome measure would similarly require new short-term health quality indicators for each procedure, and TISS and PQS could be used for other types of surgery, if the list of variables was adapted for the procedure. The I-FEED measure is strongly based on the clinical outcome of ileus, which is less relevant to many types of surgery and would require extensive adaptation.

Surgical composite quality measures can be conceptualized along a sliding scale (*Fig. 2*) where the optimum balance between clinical and administrative inputs varies according to the purpose of assessment. Traditional evaluations of surgical quality largely focus on the left side of this sliding scale, with mainly clinical inputs. The present analysis suggests that prioritization of the development and validation of composite quality measures towards the right of this balance is more likely to deliver a cost-effective tool that can act as a signal for population-level changes that affect surgical quality.

## Discussion

This review identified 10 unique composite measures that evaluate the quality of abdominal surgery at a hospital or population level. Each measure combines various data into a single score but the degree to which administrative and clinical data influences individual composite measure differs in important ways. Some, like DAOH^21^, HARM^25^ and MTL^27^ rely wholly on administrative data. Others require not just clinical data but also clinical knowledge and interpretation to a variable extent, ranging from basic (HARM^25^, MTL^27^, DAOH^21^), to intermediate (TO^28^, PQS^31^, DIMICK^32^) to expert (SCOUT^26^, I-FEED^22^, TISS^30^) levels. Consequently, the degree to which each composite quality measure is generalizable and reproducible varies. Complex inputs, especially those requiring clinical judgement, increase cost when a composite measure is implemented at scale. This review identified variable methodological rigour in the design and testing of composite measures; no measure demonstrated marked superiority, although some^25,32^ were better than others^27,28^. Taking all factors into consideration, this systematic review supports previous descriptions of challenges in the development and use of composite quality measures^11^, especially outside of a research context, but identifies a small number of composite measures that warrant further study in larger populations^21,25,27^.

Many composite measures of surgical quality rely upon a small range of input variables. Mortality is uncommon after surgery and does not always discriminate between low-quality and high-quality care but nonetheless is important^2,23^. Morbidity is similarly important, but population-level comparison is difficult as adjusting for case-mix remains a challenge. Lower performing hospitals or clinicians may fail to adequately recognize postoperative morbidity rate, erroneously resulting in apparently better performance^35^. The evaluation of quality in the surgical literature largely prioritizes technical outcomes of specific procedures, complications of treatment and the impacts of surgery on disease^2^. Such focus improves surgical care but risks overlooking population-level impacts on the quality of surgical services. In keeping with principles of measurement for improvement^4^, using a composite measure to track changes over time in a hospital or health system reduces the impact of case-mix. It is noteworthy that all composite measures evaluated in this review prioritize benchmarking or comparisons between organizations, instead of improvement over time.

The emerging inclusion of process variables in composite quality measures for surgery is notable. Length of stay varies between hospitals and health systems (and was one of the reasons for removing it from the original Clavien–Dindo score for surgical complications^2^) but is more consistent within each organization, so its use in time-series analysis for the purpose of improvement remains valid. Other variables, like readmission, are included on the assumption that it is not possible to improve these metrics without providing a higher quality of overall care^25^. Assessing the quality of surgery also requires consideration of value for money and of patient-reported outcomes and experiences but these factors are less generalizable, costly to measure and difficult to interpret on a national level^36^.

The limitations of composite measures for evaluation of surgical quality at a population level are clear: population-level databases may not capture all complications and non-fatal outcomes^21^, scores may be more useful as research tools than clinical tools^22^, small sample sizes and low event rates reduce reliability^32^, and risk and case-mix adjustment may be insufficient^10^. Although Delphi surveys and expert consensus were employed during instrument design, decisions regarding relative weighting of inputs often lacked transparency. Most measures were designed in health systems that are not publicly funded and the cost of data collection is often absent. The search criteria were defined to identify composite measures suitable for use on population data sets and may not be applicable to other contexts. Additionally, this review was confined to the English language and may exclude important perspectives.

Despite the challenges, measuring the overall quality of a surgical system of care should be an important priority for health systems as it provides an important feedback loop for day-to-day management decisions like staffing, as well as wider policy choices, like resource allocation and centralization of services^1^. A validated composite measure could act as an important safety net to support regional and remote surgical services, especially if it is generated in a cost-effective manner. Simple quality indicators that require few additional resources or training and that can be obtained from routinely collected administrative data could add real value^21,23–25,27,33^, especially if they can detect changes in surgical quality over time. On the basis of this review, further evaluation of DAOH, HARM and MTL using larger population-based data sets is recommended^21,25,27^ to test their suitability for use as a composite quality measure of abdominal surgery at a population level.

## Supplementary Material

zrad082_Supplementary_Data

## Data Availability

All data generated or analysed during this study are included in this article and the supplementary files. Code availability: software application: Rayyan AI™.
