# Supplementary material for: Composite quality measures of abdominal surgery at a population level: systematic review
Source: BJS Open. 2023 Nov 1;7(6):zrad082. doi: 10.1093/bjsopen/zrad082 (PMC10627522; doi:10.1093/bjsopen/zrad082)
Supplement: zrad082_Supplementary_Data [file zrad082_supplementary_data.zip › Supplementary_Materials.docx]

**Composite measures of abdominal surgical quality at population-level: a systematic review**

**Authors:**

Joel Rajesh^1^, Healthcare Outcomes Research Centre (HORC), Royal College of Surgeons in Ireland (RCSI), Beaux Lane House, Mercer Street Lower, Dublin, Ireland. ORCID: 0000-0001-6854-642X

Jan Sorensen^1^, Healthcare Outcomes Research Centre (HORC), Royal College of Surgeons in Ireland (RCSI), Beaux Lane House, Mercer Street Lower, Dublin, Ireland. ORCID: 0000-0003-0857-9267

Deborah A. McNamara^2^, National Clinical Programme in Surgery (NCPS), Royal College of Surgeons in Ireland (RCSI), 2 Proud’s Lane, Dublin, Ireland. ORCID: 0000-0003-3975-0485

^1^Healthcare Outcomes Research Centre, RCSI

^2^National Clinical Programme in Surgery, RCSI

**Corresponding author:**

Deborah A. McNamara^2^, National Clinical Programme in Surgery (NCPS), Royal College of Surgeons in Ireland (RCSI), 2 Proud’s Lane, Dublin, Ireland. **ORCID: 0000-0003-3975-0485**. **Twitter: @dmcsurg**

**Supplementary Materials – Index**

| **Supplementary Appendixes** |  |
| --- | --- |
| Box, search strategy terms used | *pag. 2* |
| Box, inclusion/exclusion criteria | *pag. 3* |

**Supplementary Appendixes**

Box 1. Search strategy terms used.

| Set 1  (Digestive System Surgical Procedures[Mesh]) or ("Abdomen/surgery"[Mesh]) or (surgery[Title/Abstract]) *OR*  (surgical[Title/Abstract]) and (abdom*[Title/Abstract]) or (gastrointestinal[Title/Abstract]) or (colorectal[Title/Abstract]) or (hepatobiliary[Title/Abstract])  Set 2  ("outcome score"[Title/Abstract]) or ("harm score"[Title/Abstract]) or (assess*[Title/Abstract]) or (tool*[Title/Abstract]) or (score[Title/Abstract]) and (qualit*[Title/Abstract]) or (QA[Title/Abstract])  *OR*  ("Quality Assurance, Health Care"[Mesh])  Set 3  ("Patient Outcome Assessment"[Mesh]) or ("Patient Readmission"[Mesh]) or ("Length of Stay"[Mesh]) or ("Hospital Mortality"[Mesh]) or ("Patient Discharge"[Mesh]) or ("Postoperative Complications"[Mesh]) |
| --- |

Box 2. Inclusion/Exclusion Criteria.

| **Inclusion** | **Exclusion** |
| --- | --- |
| - The primary aim of the study should be developing or improving a surgical outcome quality measurement instrument. - Includes studies with adult participants only (18 years old+) - Includes studies published as full text only - Includes studies involving abdominal and/or gastrointestinal surgery | - Not published in the English language - Not accessible or available online - Using quality assessment scores preoperatively or during the procedure. - Not relating to surgery on human patients - Using primarily patient-reported outcome measures - Have a primary focus on procedures which are not related to abdominal/ gastrointestinal surgery |
